# Breviscapine reverses doxorubicin resistance in breast cancer and its related mechanisms

**DOI:** 10.1111/1759-7714.15072

**Published:** 2023-08-16

**Authors:** Weijiang Fu, Jie Song, Haiying Li

**Affiliations:** ^1^ Department of Geriatric Medicine & Shandong Key Laboratory Cardiovascular Proteomics Qilu Hospital of Shandong University Jinan China; ^2^ Department of Medical Insurance Qilu Hospital of Shandong University Jinan China; ^3^ Department of Ultrasound Qilu Hospital of Shandong University Jinan China

**Keywords:** apoptosis, breast cancer, breviscapine, doxorubicin, P‐gp

## Abstract

**Background:**

Based on the effect of breviscapine (BRE) on reversing drug resistance of human breast cancer cell line MCF‐7/doxorubicin (Dox), the mechanism was preliminarily explored.

**Methods:**

The methyl thiazolyl tetrazolium (MTT) method was introduced to detect inhibitory effect of Dox alone or in combination with BRE on MCF‐7 (M) and MCF‐7/Dox (MD) cells, and the inhibitory concentration (IC_50_) was obtained. Cell apoptosis and Dox concentration was assessed by flow cytometry. The drug resistance multiple and reversal fold were calculated. Western blot was performed to evaluate the expression of Bcl‐2, Bax, EGFR, p‐EGFR, P‐gp, caspase‐3, and cleaved‐caspase‐3 protein in cells. The efflux of Rho‐123 was measured by flow cytometry and fluorescence microscopy.

**Results:**

The IC_50_ of Dox on MD and M cells was 16.67 and 0.71 μg/mL, respectively, with a drug resistance ratio of 23.48 times. The IC_50_ of Dox combined with BRE on MD cells was 5.62 μg/mL, with a reversal ratio of 2.97 times. BRE greatly enhanced Dox‐induced apoptosis of MD cells. Bax and cleaved‐caspase‐3 (proapoptotic protein) expression were obviously increased, while Bcl‐2 (antiapoptotic protein) expression was significantly decreased after BRE treatment. BRE inhibited EGFR activation and P‐gp expression. BRE increased the intracellular accumulation of Dox in MD cells by P‐gp.

**Conclusion:**

BRE could increase the MD sensitivity to Dox via increasing Bax and cleaved‐caspase‐3 expression and inhibiting Bcl‐2 expression, thereby promoting cell apoptosis. BRE reversed Dox resistance of MD cells by increasing Dox intracellular accumulation through inhibiting P‐gp expression via EGFR.

## Graphical abstract

The graph illustrates the possible mechanism of doxorubicin resistance in MCF‐7/Dox cells by BRE. BRE could increase the MD sensitivity to Dox via increasing Bax and cleaved‐caspase‐3 expression and inhibiting Bcl‐2 expression, thereby promoting cell apoptosis. BRE reversed Dox resistance of MD cells by increasing Dox intracellular accumulation through inhibiting P‐gp expression via EGFR. Dox, doxorubicin; BRE, breviscapine; IC_50_, half‐maximal drug inhibitory concentration; EGFR, epidermal growth factor receptor; P‐gp: P‐glycoprotein.

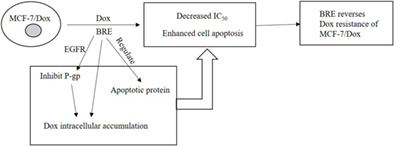

## INTRODUCTION

Breast cancer is the second leading cause of cancer death and one of the most common malignancies in women.^1^ With the continuous progress of diagnosis and treatment technology in recent years, earlier cancer diagnosis and more effective cancer treatment have been achieved, and the mortality rate of breast cancer has been significantly reduced.^2^ Current treatment strategies for breast cancer include surgery treatment and a combination of various adjuvant therapies.^3^ Most patients have a good response to the initial treatment for a period of time, but as treatment progresses, the tumor will evolve into a more aggressive type that is resistant to conventional radiotherapy and chemotherapy, eventually leading to disease recurrence, which is also the main problem facing current cancer treatment.^4^

Chemotherapy is considered to be an effective treatment for breast cancer. However, the appearance of multiple drug resistance (MDR) leads to chemotherapy failure, which is easy to cause recurrence and metastasis, and reduces the clinical efficacy of many anticancer drugs, such as doxorubicin (Dox). One of the methods to overcome MDR is to use a reversal agent. However, the application of reversal agents in clinical practice is difficult because of their poor bioavailability or serious adverse reactions.^5^ To find an effective and low toxicity drug resistance reversion agent is a research hotspot in breast cancer treatment.

Antitumor drugs extracted from natural plants are not only an important component of the modernization of traditional Chinese medicine (TCM), but also a hot topic in basic clinical medical research. Breviscapine (BRE) is the total flavonoid components extracted from the dried whole plant of Erigeron breviscapus (Vant.) Hin‐mazz (Aster breviscapus Vant.), and its main active ingredient is scutellarin.^6^ Studies have confirmed that BRE has significant antitumor effects in a variety of cancers, including prostate cancer, colorectal cancer, liver cancer, gastric cancer and other malignant tumors.^7^, ^8^ Nevertheless, the role of BRE in breast cancer therapy has rarely been elucidated. In this study, BRE from natural plants was selected to explore the resistance of breast cancer to Dox. This study provides a theoretical basis for exploring the application of BRE in reducing breast cancer drug resistance.

## METHODS

### Cell culture

Human breast cancer cells (MCF‐7; M) and their drug‐resistant cells (MCF‐7/Dox; MD) were routinely cultured and preserved in the laboratory. M cells were cultured in Dulbecco's modified eagle's medium (DMEM: HyClone) (composition: 10% fetal bovine serum [FBS: HyClone], 100 U/mL penicillin and 100 μg/mL streptomycin [Sigma‐Aldrich]). MD cells were continuously cultured in the above medium containing 1.0 μg/mL doxycycline (Dox: Sigma) to maintain cell resistance. All cells were incubated in a 5% CO_2_ cell incubator at 37°C, and the logarithmic growth cells were selected for experiment.

### Cell transfection

PCDNA3.1 and PCDNA3.1‐P‐gp was purchased from Shanghai Shenggong Biotechnology. MD cells were inoculated into six‐well plates with 3 × 10^4^ cells/well and cultured overnight. Transfection was performed with Lipofectamine 2000 (Invitrogen) according to the manufacturer's instructions. The transfection efficiency was detected by Western blot (WB).

### MTT assay was used to detect the cytotoxicity of BRE

Cells in logarithmic growth were inoculated into 96‐well plates (1 × 10^5^ cells/well). Then, different concentrations of BRE were added to make the final concentrations of 5, 10, 20, 40, and 80 μg/mL, respectively. An equal volume of DMEM medium was added to the control group. After incubation for 24 h, 20 μL methyl thiazolyl tetrazolium (MTT: Sigma) was added. After continued culture for 4 h, the supernatant was removed and 150 μL/well dimethyl sulfoxide solution (DMSO: Sigma) was added. After oscillating for 10 min with a microwave oscillator, the crystals were completely dissolved. Absorbance value (OD_490 nm_) of each well was detected by a microplate spectrophotometer (Promega). Each group was repeated for six wells, and growth inhibition rate (IR) was calculated.

Inhibition rate (IR) = (1‐OD value of drug group/OD value of control group) × 100%.

### IC_50_ of Dox against breast cells and drug resistance multiple was calculated

Cells in logarithmic growth were inoculated into a 96‐well plate (1 × 10^5^ cells/well). 10^−2^, 10^−1^, 10^0^, 10^1^ and 10^2^ μg/mL Dox was added to each experimental group, and the experimental method was the same as detailed above.

Resistance ratio = IC_50_ value of resistant cells/IC_50_ value of sensitive cells.

### Reversal effect of BRE on drug resistance of MD cells

Cell culture and experimental methods were the same as detailed above. Dox in different concentrations was added, and the OD value was measured. The changes in the killing effect of Dox on MD cells were observed after noncytotoxic BRE was combined with Dox. The IC_50_ of BRE on MD cells after the combination of Dox with noncytotoxic concentration BRE and the reversal fold was calculated.

Reversal fold (RF) = prereversal IC_50_ value/post‐reversal IC_50_ value.

### Cell apoptosis was assessed by flow cytometry

Cells were inoculated in a 24‐well plate (5 × 10^4^ cells/well). Dox and BRE treated cells alone or in combination. An equal volume of DMEM medium was added to the negative control group. The cells were collected after 24 h of culture and centrifuged at 251 *g* to remove the supernatant. The cells were resuspended with phosphate buffered saline (PBS). Annexin V‐FITC was added into the cell suspension (5 × 10^6^ cells) and mixed gently. After 10 min in the dark, propidium iodide (PI) was added. After mixing, the cells were incubated at room temperature in the dark for 10 min. Apoptotic cells were detected by flow cytometry. The experiment was repeated three times.

### Dox concentration was determined by flow cytometry

M cells and MD cells in logarithmic growth were seeded into a 24‐well plate with 5 × 10^4^ cells/well and cultured in a cell incubator for 24 h (density to 80%). Cells were divided into two groups: Dox only was added to one group, and Dox and BRE were added to the other group. After incubation at 37°C for 2 h, the cells were washed twice with precooled PBS. After trypsin digestion, the cell suspension was collected and run through a flow cytometer. The fluorescence intensity of Dox excitation, representing the intracellular Dox concentration, was measured by flow cytometry.

### Rhodamine 123 (Rho‐123) efflux test

The cell suspension of M and MD were digested with trypsin. Cells were seeded at 4 × 10^5^ per well on six‐well culture plates and incubated at 37°C and 5% CO_2_ for 24 h. Then, cells were treated with BRE for 48 h. After washing and digesting cells, Rho‐123 (10 μmol/L) was added and incubated for 30 min at room temperature in the dark. At the end of incubation, the cells were washed with ice‐cold PBS and resuspended in PBS. The accumulation of Rho‐123 in cells was detected by flow cytometry and fluorescence microscope.

### Western blot

After cells were treated for 48 h, total protein was extracted with 100 μL of radio immunoprecipitation assay (RIPA) lysate (Hyclone‐Pierce) containing 1% protease inhibitor. Cells were transferred into Eppendorf (EP) tubes and centrifuged at 13 400 *g* for 10 min to collect supernatant. Protein quantification was performed using the bicinchoninic acid (BCA) method. The appropriate amount of sodium dodecyl sulfate (SDS) loading buffer was added for cross‐linking under metal bath conditions at 100°C. The 20 μg protein sample was taken for protein separation by 10% SDS‐PAGE. Proteins separated by electrophoresis were electro‐transferred to polyvinylidene fluoride (PVDF) membranes (Millipore). The proteins were placed in 5% nonfat milk blocking solution and sealed for 1 h at room temperature. The primary antibody (actin, EGFR, p‐EGFR, P‐gp, Bcl‐2, Bax, caspase‐3, or cleaved‐caspase‐3 antibody; 1:1000) was added and incubated overnight at 4°C. The horseradish peroxidase (HRP)‐conjugated secondary antibody (1:5000) was incubated at room temperature for 1 h. After washing the membrane, bands were visualized by enhanced chemiluminescence (ECL: Millipore).

### Statistical analysis

Statistical analysis was carried out using Graphpad prism 6 or SPSS 17.0 software. All data are represented by mean ± standard deviation ($\bar{x}\pm s$). One‐way ANOVA or *t*‐test was used for comparison between groups. *p <* 0.05 was considered statistically significant.

## RESULTS

### Inhibition of BRE on the proliferation of M and MD cells

**FIGURE 1:**
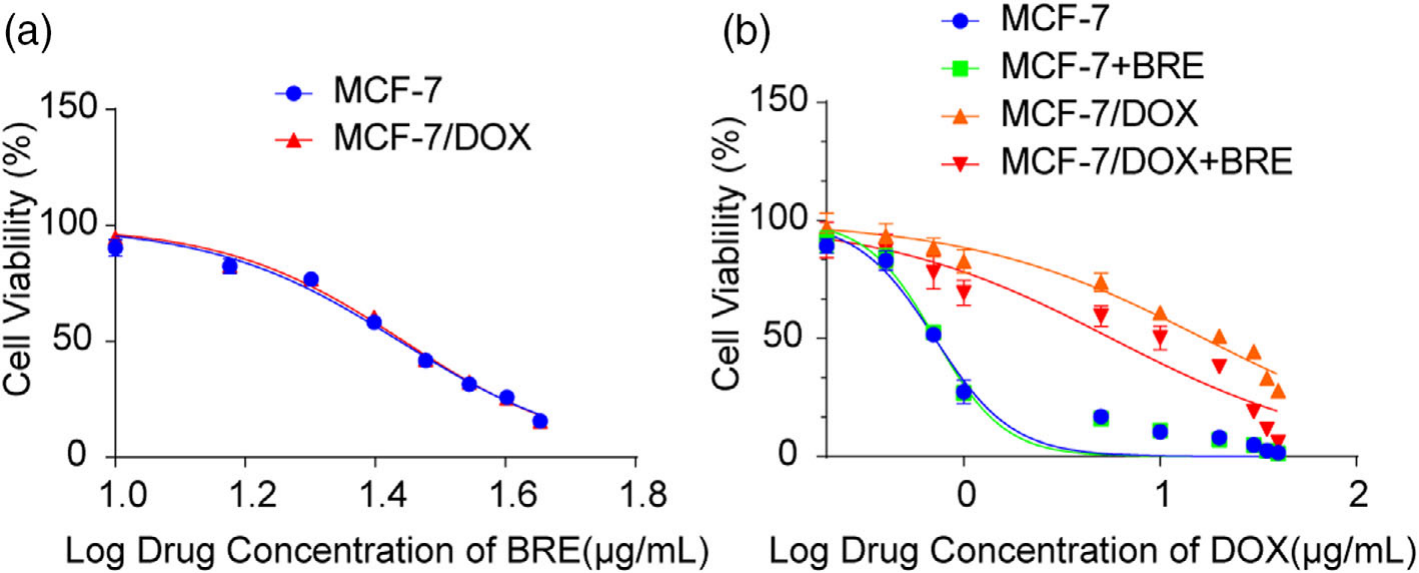
Breviscapine (BRE) inhibited the proliferation of MCF‐7 (M) and MCF‐7/Dox (MD) cells and reversed the drug resistance of MD cells. (a) Effect of treatment with different concentrations of BRE on the viability of M and MD cells. (b) Effect of BRE combined with Dox on the viability of M and MD cells.

MTT cytotoxicity assay was performed to detect the cytotoxicity and reversal activity of BRE on breast cancer cells. The results showed that BRE inhibited the proliferation of M and MD cells in a dose‐dependent manner (Figure 1a). BRE had inhibitory effects on the proliferation of M and MD cells (Figure 1a). When BRE concentration was less than 10 μg/mL, the inhibition rate of BRE on M and MD cells was less than 10%, and there was no cytotoxicity (Figure 1a). Therefore, 10 μg/mL of BRE was selected as the noncytotoxic dose and reversing agent concentration.

### Reversal effect of BRE on drug resistance of MD cells

The results showed that the IC_50_ of Dox for M and MD cells was different (Figure 1b). The IC_50_ of Dox for M cells was 0.71 μg/mL and IC_50_ for MD cells was 16.67 μg/mL, and the drug resistance ratio was 23.48. The IC_50_ of Dox on MD cells was decreased after the combination of Dox and BRE without cytotoxic concentration (10 μg/mL) on MD cells (Figure 1b). The IC_50_ of Dox on MD cells was 5.62 and the reversal fold was 2.97 when 10 μg/mL of BRE was used as the reverse agent. BRE could partially reverse the drug resistance of MD cells in drug‐resistant human breast cancer cells.

### BRE enhanced Dox‐induced apoptosis in MD cells

**FIGURE 2:**
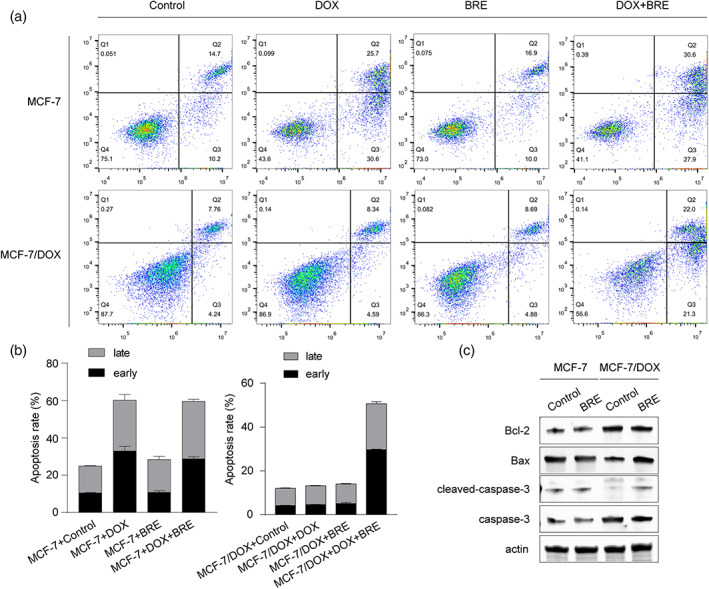
Breviscapine (BRE) enhanced cell apoptosis induced by Dox in MCF‐7/Dox (MD) cells. (a, b) The effect of Dox and BRE on apoptosis of MCF‐7 (M) and MD cells was detected by flow cytometry. (c) Western blot was used to detect the effect of BRE on apoptosis‐related proteins in M and MD cells.

Inducing cancer cell apoptosis is a common mechanism of action among many antitumor drugs. BRE treatment alone did not significantly affect the apoptosis level in either M or MD cells compared with control (Figure 2a,b). The apoptosis rate of MD cells treated with BRE and Dox in combination was higher than that treated with Dox or BRE alone (Figure 2a,b). However, there was no significant difference in the apoptosis rate of M cells treated with BRE and Dox compared to those treated with Dox alone (Figure 2a,b). These results indicated that BRE greatly enhanced Dox‐induced apoptosis in drug‐resistant breast cancer cells.

### Effects of BRE on apoptosis‐related protein levels in MD cells

The effect of 10 μg/mL BRE on apoptosis‐related protein expression in M and MD cells was detected by WB. Compared with the control group, BRE treatment significantly increased the expression of proapoptotic proteins Bax and cleaved caspase‐3, and significantly decreased the expression of antiapoptotic protein Bcl‐2 (Figure 2c). Inducing apoptosis may be one of the mechanisms of BRE reversing drug resistance of MD cells.

### BRE increased the intracellular accumulation of Dox in MD cells

**FIGURE 3:**
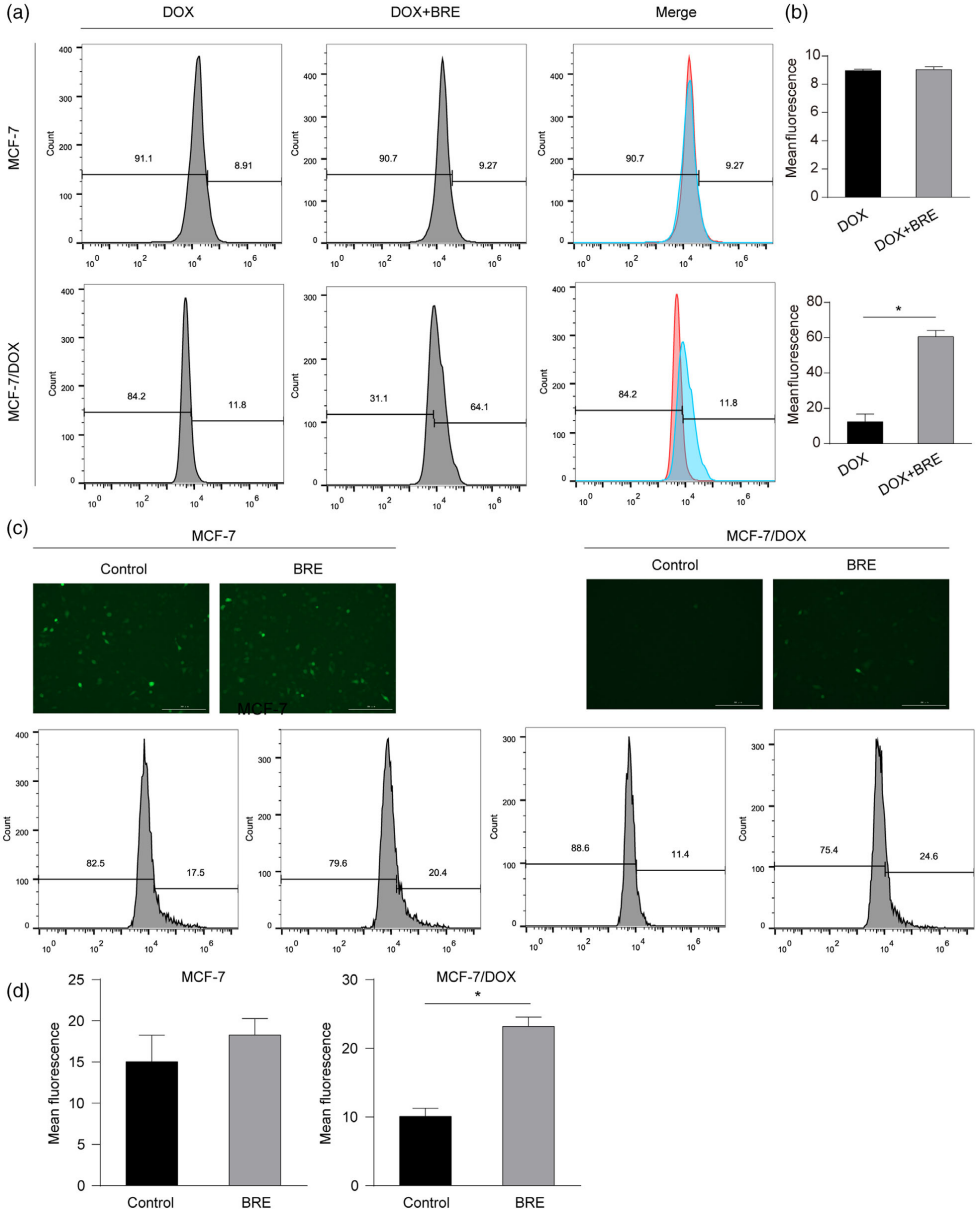
Effect of breviscapine (BRE) treatment on the amount of intracellular Dox in MCF‐7 (M) and MCF‐7/Dox (MD) cells. (a, b) The intracellular Dox accumulation of M and MD cells was detected by flow cytometry. (c, d) The intracellular Rho‐123 accumulation of M and MD cells was detected by fluorescence microscopy and flow cytometry. *p < 0.05, compared with control.

To investigate whether BRE can reverse drug resistance by increasing intracellular Dox concentration, flow cytometry was used to detect the fluorescence intensity to determine the intracellular Dox concentration in cells with or without BRE. Dox alone showed only weak fluorescence intensity in resistant MD cells compared to sensitive M cells (Figure 3a,b). However, stronger fluorescence intensity was detected in the combination of BRE and Dox compared with Dox alone in MD cells (Figure 3a,b). When BRE was combined with Dox in M cells, the fluorescence intensity did not change significantly compared with Dox alone (Figure 3a,b). These results indicated that BRE increased the intracellular accumulation of Dox in drug‐resistant cells, thereby increasing the cytotoxic effect of Dox by reversing MDR.

Rhodamine, as the transport substrate of P‐gp, can better represent its transport function.^9^ Subsequently, changes in the efflux of P‐gp substrate reagent Rho‐123 in cells with or without BRE were detected by fluorescence microscope and flow cytometry. In the presence of BRE, the intracellular level of Rho‐123 in MD cells was significantly increased compared with control (Figure 3c,d). However, the intracellular accumulation of Rho‐123 did not change in the presence of BRE in M cells (Figure 3c,d). These results indicated that BRE may reverse MDR by reducing drug efflux and increasing Dox intracellular accumulation in MD cells through P‐gp. However, this requires further experimental verification.

### BRE reduced P‐gp expression by inhibiting EGFR, thereby reversing the drug resistance of cells

**FIGURE 4:**
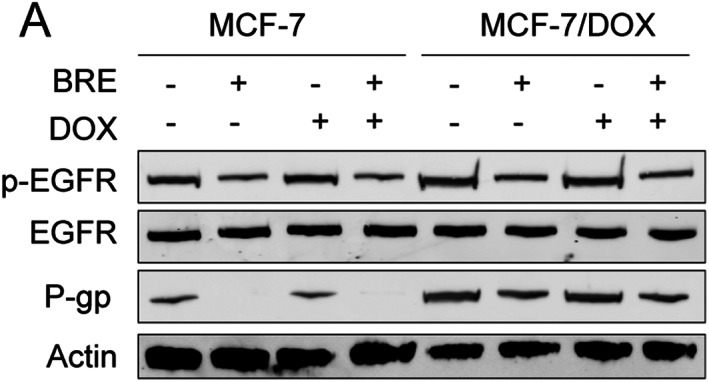
Epidermal growth factor receptor (EGFR), phospho‐epidermal growth factor receptor (p‐EGFR) and P‐glycoprotein (P‐gp) protein expression levels in MCF‐7 (M) and MCF‐7/Dox (MD) cells after cotreatment with breviscapine (BRE) and doxycycline (Dox).

In order to verify whether BRE changes P‐gp expression, the expression of P‐gp and its upstream pathway key molecule EGFR was detected by WB. Compared with M cells, P‐gp expression in MD cells was significantly increased, and EGFR was over‐activated (Figure 4). BRE decreased the expression of P‐gp protein and inhibited the activation of EGFR in both M and MD cells (Figure 4).

**FIGURE 5:**
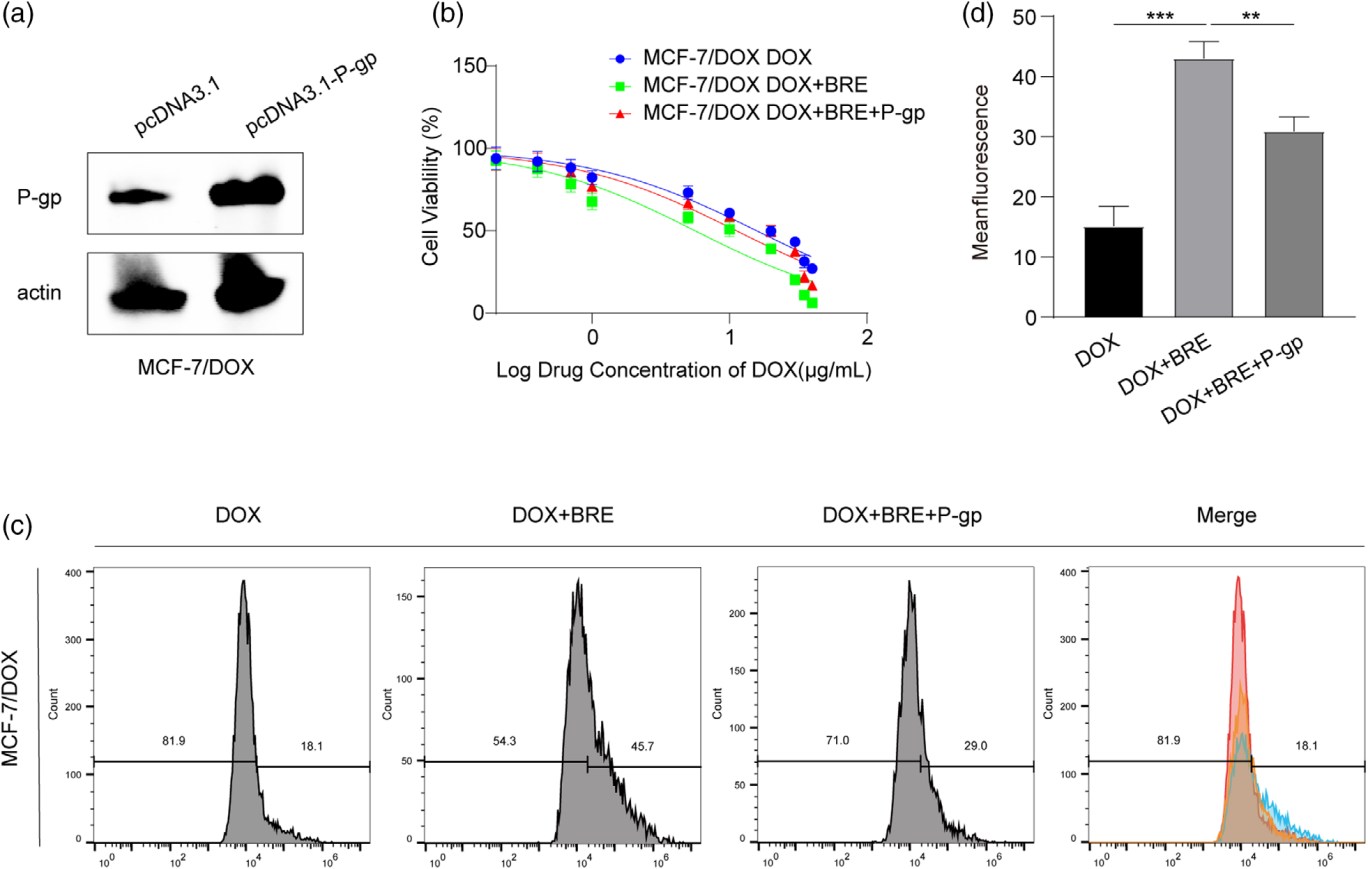
Breviscapine (BRE) reversed multiple drug resistance (MDR) by increasing intracellular drug accumulation through P‐gp. (a) The transfection efficiency of PCDNA3.1‐P‐gp in MD cells was detected by Western blot. (b) Effect of P‐gp overexpression on the viability of MD cells treated by doxycycline (Dox) and BRE. (c, d) P‐gp overexpression could reverse the effects of BRE treatment on Dox accumulation in MD cells. **p < 0.01, ***p < 0.001, compared with Dox or Dox + BRE group.

To further verify that BRE reversed MDR by reducing drug efflux and increasing intracellular drug accumulation through P‐gp, P‐gp was overexpressed in MD cells (Figure 5a). After overexpression of P‐gp, the IC50 (11.23 μg/mL) of MD cells was increased versus Dox + BRE group (5.62 μg/mL) (Figure 5b). Moreover, flow cytometry results showed that P‐gp overexpression reduced intracellular Dox concentration in MD cells versus Dox + BRE group (Figure 5c,d). These results indicated that BRE reversed drug resistance by inhibiting P‐gp expression through EGFR.

## DISCUSSION

Clinically, chemotherapy, radiotherapy and surgery are the main methods for treating tumors.^10^ With the development and application of chemotherapy, chemotherapy resistance has become a difficulty problem in tumor therapy.^11^ Reversing drug resistance plays a vital role in improving chemotherapy efficacy of drug‐resistant tumors. There are abundant resources and extensive pharmacological effects of TCM, and many of which themselves have anticancer effects, suggesting that screening reversal agents in TCM has great advantages. Although in vitro experiments have proved that many drugs can reverse the drug resistance of tumor cells, only a few drugs have entered clinical studies, and their clinical efficacy is not ideal.^12^ In recent years, searching for new drug resistance reversal agents from TCM has become a hot topic.

BRE has been reported to have significant antitumor effects. Wu et al. found that BRE could induce apoptosis of HepG2 cells and could inhibit HepG2 activity in vitro, and it was preliminarily believed that the inhibitory effect was related to the change in Bcl‐2, Bax and caspase‐3 expression.^7^ BRE exerts antitumor and antimetastasis roles by regulating the PAQR4‐mediated PI3K/Akt pathway in prostate cancer.^13^ Moreover, in another study, BRE induced growth inhibition and apoptosis of A549 cells by mediating miR‐7 expression.^14^ Other studies show that BRE has antiproliferative effects on breast cancer cells, and can prevent recurrence and metastasis of breast cancer.^15^ This study mainly observed the reversal effect of BRE on breast cancer resistance. The results showed that MD cells had strong resistance to Dox, and the combination of BRE could effectively reverse the resistance. The BRE regulated the expression of proteins (Bcl‐2, Bax, and cleaved‐caspase‐3) in the apoptosis pathway, thus increasing the sensitivity of MD cells to Dox and promoting the apoptosis. It suggested that BRE can reverse the resistance of MD cells to Dox, possibly by regulating the expression of apoptosis proteins, improving the sensitivity of MD cells to Dox, and promoting the apoptosis of breast cancer cells.

One of the mechanisms of MDR is the reduction of intracellular drug concentration, resulting in hyposensitivity to drugs, in which enhanced drug efflux plays an important role.^16^ The overexpression of ATP‐binding cassette (ABC) cell membrane transporter P‐gp can increase drug efflux and reduce the concentration of intracellular chemotherapy drugs, which mediates the occurrence of MDR.^17^ Abnormal expression and activation of EGFR which belongs to the tyrosine kinase family, can lead to chemotherapy resistance of tumor cells.^18^ Drug resistance in tumor cells is increased by EGFR overexpression.^19^ Studies have found that the expression of P‐gp may be regulated by the EGFR signaling pathway.^20^, ^21^ The results of this study showed BRE reversal resistance was reversed after P‐gp overexpression. Moreover, BRE could reverse drug resistance by reducing P‐gp and reducing drug pumping out of cells and decrease the expression of phospho‐epidermal growth factor receptor (p‐EGFR) and P‐gp in MD cells after Dox treatment. It is suggested that BRE reversed drug resistance of breast cancer through EGFR inhibition of P‐gp expression.

In conclusion in this study, it was found that BRE could affect the drug resistance of drug‐resistant breast cancer cells at the cellular level. Moreover, this function may be achieved by the effect of BRE on the expression level and the transport ability of P‐gp protein through EGFR. As an effective drug resistance reversion agent, the mechanism of BRE reversing MDR and its safety and effectiveness in in vivo application deserve further research.

## AUTHOR CONTRIBUTIONS

Weijiang Fu conceived and designed this study. Jie Song and Haiying Li performed the assay and interpreted the results. Haiying Li was involved in drafting the manuscript and revising it critically for important intellectual content. All authors read and approved the final manuscript.

## FUNDING INFORMATION

The author(s) received no financial support for the research, authorship, and/or publication of this article.

## CONFLICT OF INTEREST STATEMENT

The authors declare that they have no competing interests.

## Data Availability

Data to support the findings of this study is available on reasonable request from the corresponding author.
